# The anti-cancer effects of itraconazole in epithelial ovarian cancer

**DOI:** 10.1038/s41598-017-06510-7

**Published:** 2017-07-26

**Authors:** Chel Hun Choi, Ji-Yoon Ryu, Young-Jae Cho, Hye-Kyung Jeon, Jung-Joo Choi, Kris Ylaya, Yoo-Young Lee, Tae-Joong Kim, Joon-Yong Chung, Stephen M. Hewitt, Byoung-Gie Kim, Duk-Soo Bae, Jeong-Won Lee

**Affiliations:** 1 Departments of Obstetrics and Gynecology, Samsung Medical Center, Sungkyunkwan University School of Medicine, Seoul, Korea; 2 Experimental Pathology Laboratory, Laboratory of Pathology, Center for Cancer Research, National Cancer Institute, National Institutes of Health, Bethesda, MD 20892 USA

## Abstract

We assessed the anti-proliferative activity of itraconazole using an EOC cell line (SKOV3ip1) and endothelial cell lines (HUVEC & SVEC4-10). We also examined angiogenesis (VEGFR2, p-ERK, p-PLCr1/2), hedgehog (Gli1, Ptch1, SMO), and mTOR (pS6K1) signaling pathways to determine the mechanism of action of itraconazole. Furthermore, we evaluated the synergistic effects of itraconazole and paclitaxel using orthotopic mouse models with established EOC cells (SKOV3ip1 or HeyA8) as well as patient-derived xenografts (PDXs). Itraconazole treatment inhibited proliferation of endothelial cells in a dose-dependent manner, but had no effect on EOC cells. The endothelial cell antiproliferative effect was associated with inhibition of hedgehog, and mTOR pathways and angiogenesis. In xenograft models of EOC using SKOV3ip1 or HeyA8, mice treated with the combination of itraconazole and paclitaxel had significantly decreased tumor weight than the control, paclitaxel-alone, or itraconazole-alone groups. Tissue derived from these tumors had significantly lower microvessel density than tissue from the other groups as well as hedgehog and mTOR pathway inhibition. We confirmed those effects in two EOC PDX models. These results suggest that itraconazole selectively inhibits endothelial cells rather than cancer cells by targeting multiple pathways including hedgehog, and mTOR pathways and angiogenesis.

## Introduction

Epithelial ovarian cancer (EOC) is the second most common gynecologic cancer. It is the most fatal disease and the fifth leading cause of cancer death among women^1^. Patients with advanced EOC have a 5-year survival rate of only 40%^2^. The standard treatment for EOC is surgical cytoreduction followed by adjuvant combination chemotherapy. Despite improvements in surgical and chemotherapeutic approaches, the majority of women with relapsed EOC eventually die from the disease, and there is an unmet and urgent need to improve treatment.

Itraconazole, a widely used antifungal drug, has recently been shown to have anti-cancer effects against several cancer types. It significantly enhanced the antitumor efficacy of cisplatin in primary xenograft models of human non-small cell lung cancer^3^ and also inhibited tumor growth and metastasis in a prostate cancer mouse model^4^. The suggested mechanisms of its antitumor effect are inhibition of VEGFR2 and its downstream substrate^5^, target of rapamycin (mTOR)^6^, as well as inhibition of hedgehog signaling^7^. Currently, there are several active clinical trials evaluating itraconazole as a cancer therapeutic in non-small cell lung cancer (NCT02357836), esophageal cancer (NCT02749513), and basal cell carcinoma (NCT02120677). A recent study also reported that itraconazole is a feasible treatment option for metastatic basal cell carcinoma^8^.

Several emerging agents with novel targets are in development for treatment of EOC. Among those agents, the addition of bevacizumab, an antiangiogenic agent, to the standard therapy of taxane and carboplatin was shown to significantly improve progression-free survival and arguably has become the standard of care for select patients^9, 10^. However, the overall gain in survival is marginal with a high cost of maintenance. The limitations of anti-VEGF therapy have been attributed to the presence of alternative pathways in pro-angiogenic signaling networks; drugs that can target multiple pathways simultaneously are therefore required.

The purpose of this study was to test the anti-cancer effects of itraconazole on tumor development and growth in EOC models, including orthotopic cell line xenografts and patient-derived xenograft (PDX) models, and to determine the possible underlying mechanisms. This study offers the first assessment of the efficacy and mechanisms of itraconazole as an anticancer therapeutic in preclinical models of EOCs.

## Results

### Itraconazole significantly affects cell proliferation and apoptosis of endothelial cells but has no direct effects on ovarian cancer cells

**Figure 1:**
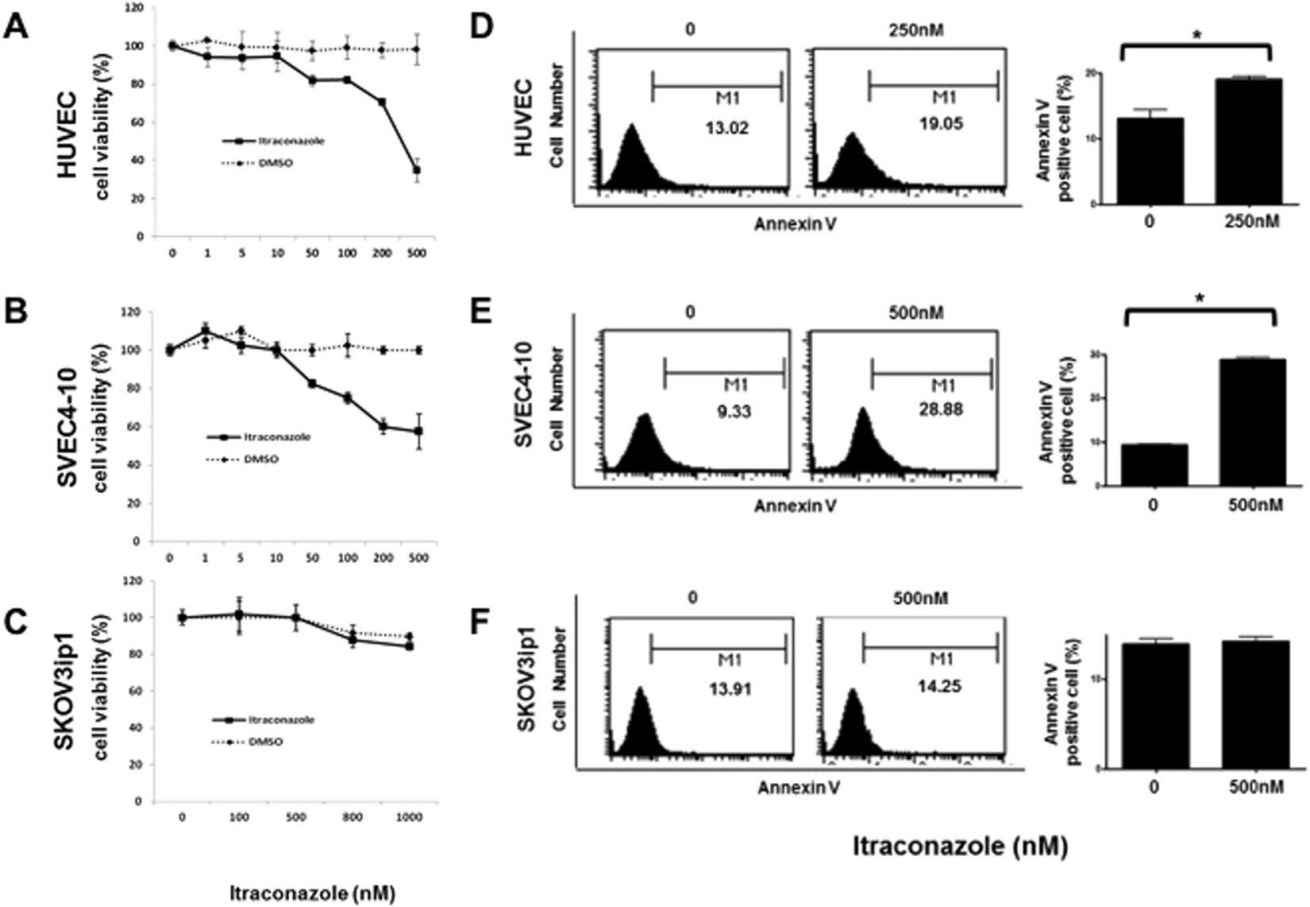
Inhibition of endothelial cell proliferation following treatment with itraconazole. Itraconazole inhibited cell proliferation in a dose-dependent manner, as evaluated by the MTT assay in HUVEC (**A**) and SVEC4-10 (**B**) cells. Proliferation of SKOV3 cells was not inhibited (**C**). Flow cytometric determination of apoptosis yielded similar results (**D–F**). The significance of differences was determined by unpaired t-test, and values of *P* < 0.05 (*) or *P* < 0.01 (**) were considered to be statistically significant.

Human (HUVEC) and mouse (SVEC4-10) endothelial cells were initially treated with itraconazole for 48 h to confirm the inhibitory activity of itraconazole on endothelial cell proliferation. Itraconazole inhibited proliferation of HUVEC and SVEC4-10 endothelial cells in a dose-dependent manner (Fig. 1A and B). To assess the relative specificity of its inhibitory activity on endothelial cells, the antiproliferative effects of itraconazole were examined in an ovarian cancer cell line (SKOV3ip1). Itraconazole had no effect on proliferation up to the maximum tested concentration (Fig. 1C). Apoptosis induction measured by annexin V-incorporation after treatment with itraconazole (250 nM for HUVEC and 500 nM for SVEC4-10) resulted in significantly greater apoptosis in itraconazole-treated endothelial cells than control cells, but not in SKOV3ip1 cells (Fig. 1D and F).

Interestingly, the antiproliferative effect of itraconazole was enhanced when combined with paclitaxel which is the main chemotherapeutic agent in ovarian cancer (Supplemental Fig. S1). The synergistic effect was not seen in SKOV3ip1 cells.

### Itraconazole inhibits VEGFR2, hedgehog, and mTOR signaling pathways in endothelial cells

**Figure 2:**
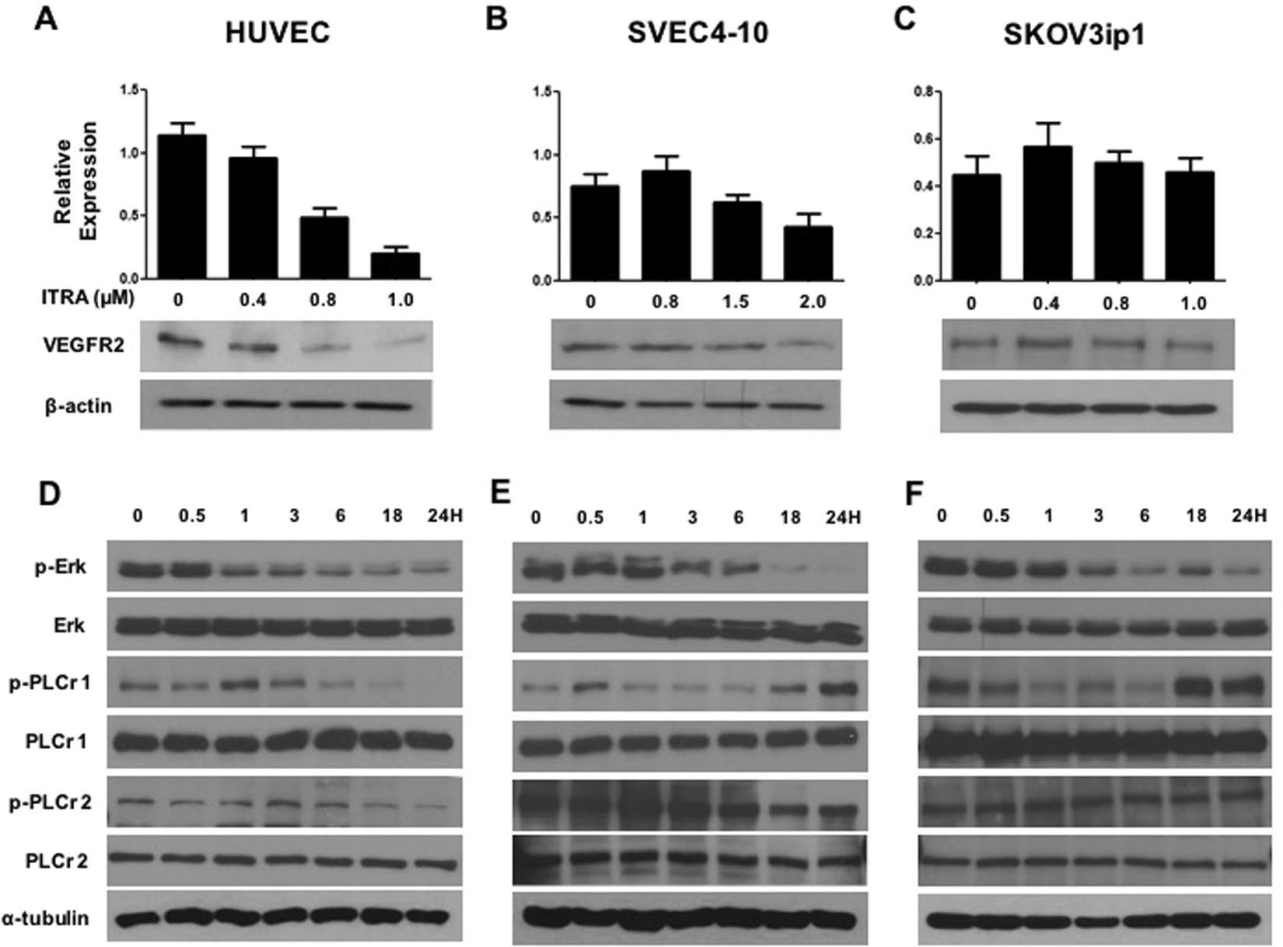
VEGF2 signaling is down-regulated by itraconazole. HUVEC (**A**), SVEC4-10 (**B**), and SKOV3ip1 (**C**) cells were treated with various doses of itraconazole, and VEGFR2 expression was determined by Western blot. Cells were treated for 24 h with the indicated doses of itraconazole and analyzed by Western blot for activating phosphorylation of VEGFR2 (Tyr-1175), PLCr1/2 (Tyr-783), and ERK1/2 (Thr-202/Tyr-204). In vehicle-treated endothelial cells, ERK phosphorylation was inhibited starting 1 h after itraconazole treatment. PLCr1 phosphorylation peaked 30–60 min after itraconazole treatment, and the signal decayed thereafter. Itraconazole did not block PLCr2 activation in either endothelial or cancer cells. ITRA, itraconazole. The gels images were cropped and full-length gels and blots are included in the Supplementary Figure S4.

Having observed that itraconazole inhibited endothelial cell growth, we next investigated whether VEGFR2 activation was perturbed. To test this, HUVEC, SVEC4-10, and SKOV3ip1 cells were incubated in the presence of varying dose of itraconazole, and VEGFR2 expression was determined by Western blotting. Treatment with itraconazole markedly decreased the expression of VEGFR2 in endothelial cells in a dose-dependent manner (Fig. 2A and B), but not in SKOV3ip1 cells (Fig. 2C). In mouse endothelial cells (SVEC4-10), the dose of itraconazole were different but showed the same observations.

**Figure 3:**
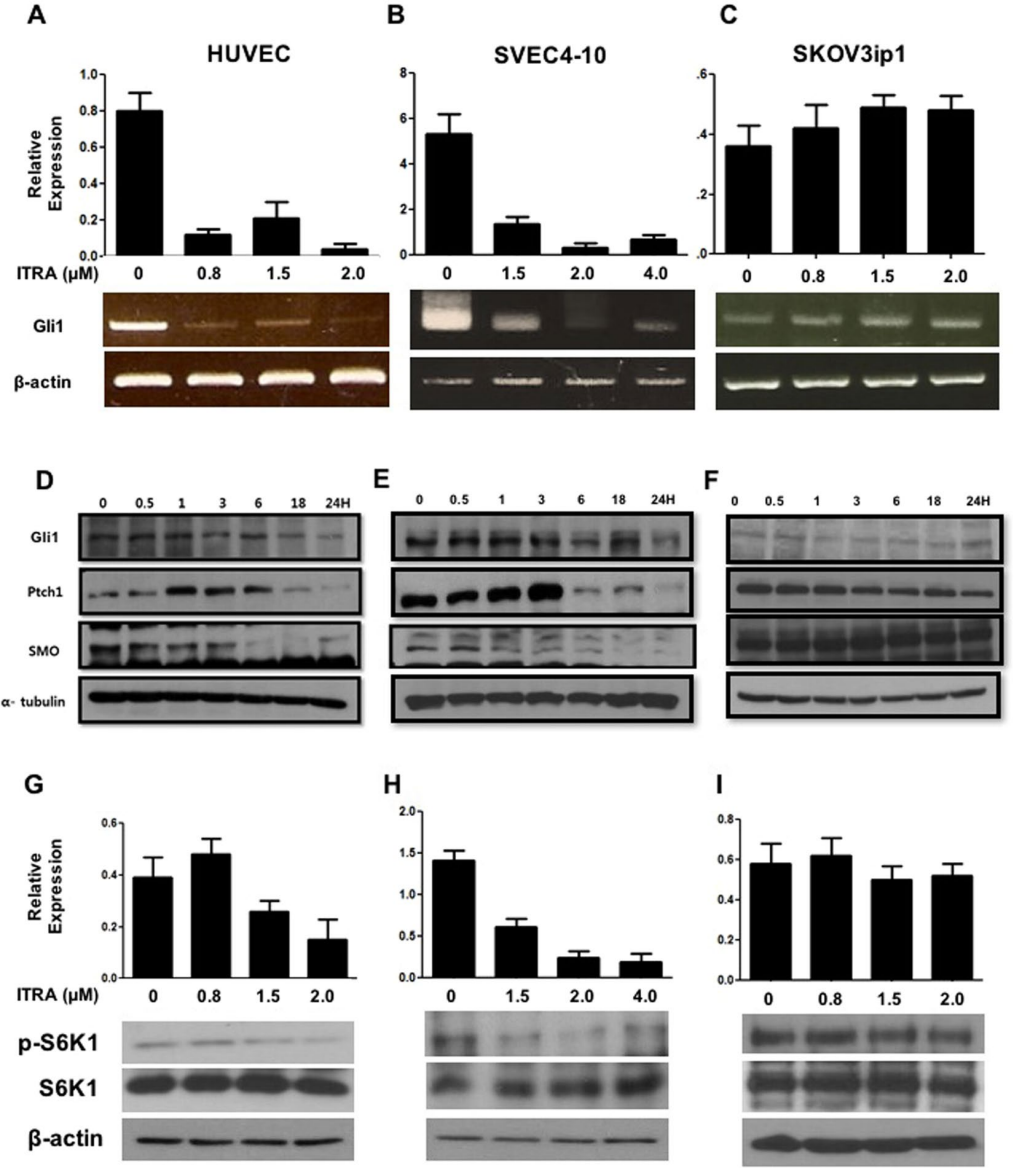
Inhibition of hedgehog and mTOR pathways by itraconazole. The expression of Gli1, Ptch1, and SMO, which are central for the activation of hedgehog signaling, was tested in HUVEC (**A** and **D**), SVEC4-10 (**B** and **E**), and SKOV3ip1 (**C** and **F**) cells. Cells were treated with ITRA and processed in the same manner as in Fig. 2. The molecules were inhibited by itraconazole in endothelial cells, but levels of these molecules did not change in SKOV3ip1 cells. mTOR inhibition was determined based on the phosphorylation status of the substrate S6 kinase (S6K) after treatment with itraconazole as determined by Western blot in HUVEC (**G**), SVEC4-10 (**H**), and SKOV3ip1 (**I)** cells. Itraconazole inhibited the phosphorylation of S6K1 in a dose-dependent manner in endothelial cells (**A** and **B**), while no inhibition was observed in ovarian cancer cells. The gels images were cropped and full-length gels and blots are included in the Supplementary Figure S5.

Phosphorylation of ERK and phospholipase C gamma 1/2 (PLCr1), a direct binding partner of VEGFR2, was also examined (Fig. 2D–F). In three cell lines, ERK phosphorylation was inhibited 1 h after itraconazole treatment (HUVEC 1 uM, SEVC4-10 and SKOV3ip1 1.5 uM). PLCr1 phosphorylation peaked 30–60 min after itraconazole, and the signal decayed thereafter. Itraconazole did not block PLCr2 activation in either endothelial or cancer cells. As itraconazole has been found to affect the hedgehog pathway^11^, we examined the expression of Gli1, Ptch1, and SMO, which are central for the activation of this pathway. Gli1 were inhibited by itraconazole in endothelial cells, but their levels remained unchanged in SKOV3ip1 cells (Fig. 3A–C). The protein levels of Gli1, Ptch1, and SMO were examined after itraconazole treatment, and signals decayed after peak in endothelial cells, which was not shown in cancer cell (Fig. 3D–F). Next, we determined the effect of itraconazole on the phosphorylation state of S6 kinase, a canonical mTOR substrate. Like rapamycin, itraconazole inhibited the phosphorylation of S6K1 in a dose-dependent manner in endothelial cells (Fig. 3G and H). Inhibition of S6K1 phosphorylation was not observed in ovarian cancer cells, suggesting specificity of inhibition in endothelial cells (Fig. 3I). Together, these results suggest that the antiangiogenic activity of itraconazole is due to its ability to inhibit the VEGFR2, hedgehog, and mTOR pathways.

To examine the change of oxidative stress, we checked the expressions of superoxide dismutase 2 (SOD2) and catalase. Supplemental Fig. S1 shows that catalase is increased with treatment of itraconazole. In addition, intracellular ROS were evaluated and we found more increased expression in endothelial cells including HUVEC and SVEC4-10 compared with EOC cells following itraconazole treatment.

### Itraconazole and paclitaxel synergistically inhibit tumor growth in cell line orthotopic xenografts of EOC

To assess the potential clinical relevance of the *in vitro* results, we performed *in vivo* experiments using EOC orthotopic mouse models. SKOV3ip1 and HeyA8 ovarian cancer cells were implanted into the peritoneal cavity of female nude mice, and from 7 days after cell injection, therapy was started according to the following four treatment regimens: control, paclitaxel, itraconazole, and co-treatment with paclitaxel and itraconazole.

**Figure 4:**
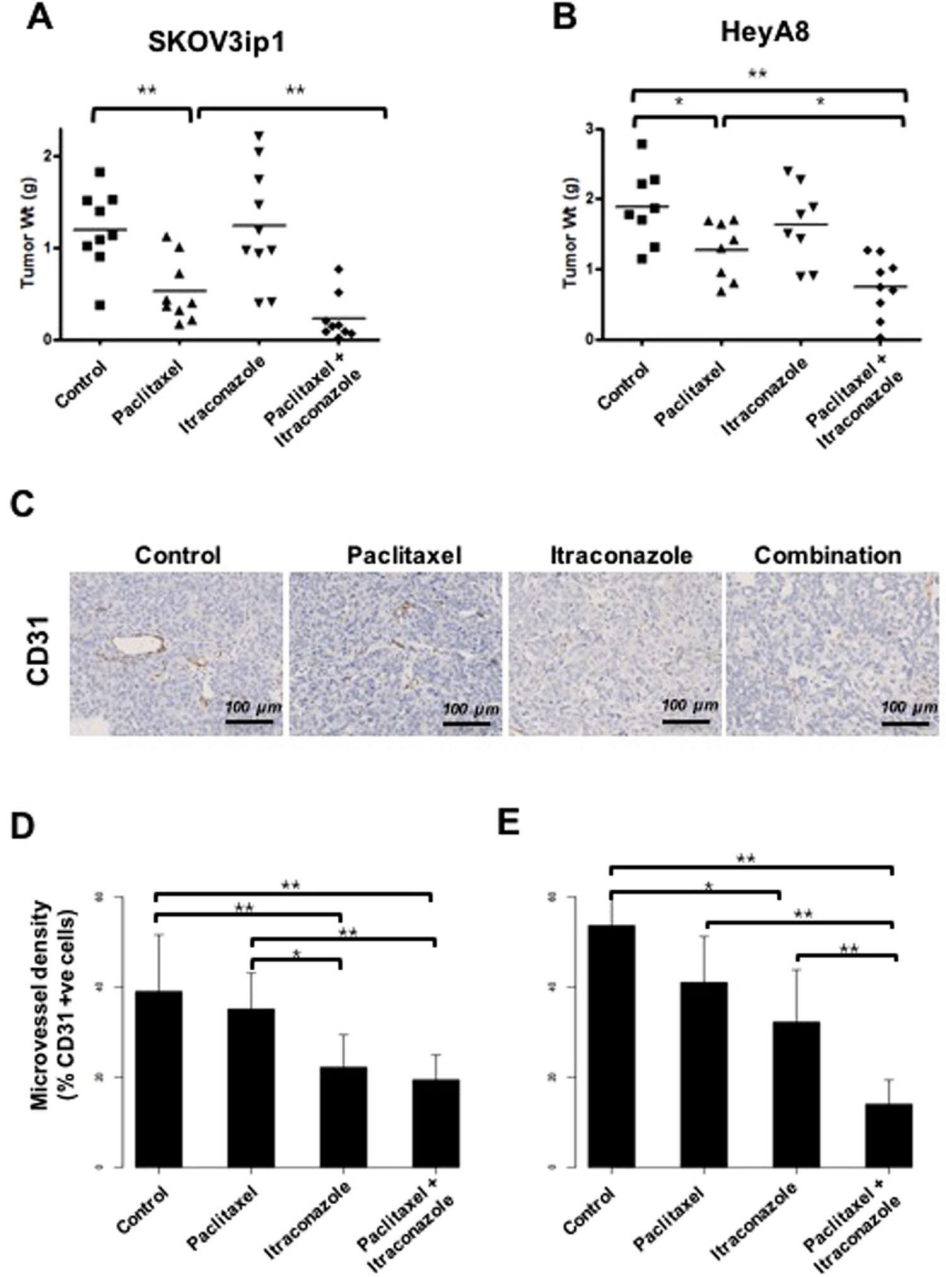
Itraconazole inhibits growth of ovarian cancer xenografts and these xenografts have decreased microvessel density. Mice bearing ovarian cancer cells were treated with vehicle (*n* = 10), oral itraconazole 20 mg/kg twice daily (*n* = 10), paclitaxel 6 mg/kg intraperitoneally every 7 days (*n* = 10), or a combination of itraconazole and paclitaxel (*n* = 10). Mice treated with paclitaxel in combination with itraconazole had significantly lower tumor weight than mice treated with paclitaxel alone (**A** and **B**). CD31-positive tumor vascular area was measured in tumors treated with vehicle (*n* = 5), itraconazole (*n* = 5), paclitaxel (n = 5), or a combination of itraconazole and paclitaxel (*n* = 5) in SKOV3ip (**C** and **D**) and HeyA8 (**E**) xenograft models. Tumors from mice that received itraconazole monotherapy had a reduced tumor vascular area compared with vehicle-treated tumors (*P* = 0.004 for SKOV3ip1 and *P* = 0.016 for HeyA8, respectively). Addition of itraconazole to paclitaxel reduced tumor vasculature from 35% to 19% for SKOV3ip1 (*P* = 0.004) and from 41% to 14% for HeyA8 (*P* < 0.001).

Itraconazole alone was not effective at inhibiting tumor growth compared with the control, while paclitaxel alone was effective. However, mice treated with paclitaxel in combination with itraconazole had significantly decreased tumor weight compared with mice treated with paclitaxel alone, suggesting that itraconazole had a synergistic effect of (*P* < 0.001 and *P* = 0.030, respectively, Fig. 4A and B). Daily monitoring of animals throughout the course of therapy showed acceptable tolerability with no untoward side effects, such as changes in body weight, mobility, posture, or feeding habits.

Based on our data that itraconazole had anti-angiogenic effects *in vitro*, we determined the microvessel density in our xenograft system. Figure 4C demonstrates representative examples of the microvasculature, with staining of endothelial cells by CD31. Analysis demonstrated the addition of itraconazole to paclitaxel reduced tumor vasculature from 41% to 14% for HeyA8 (*p* < 0.001) and from 35% to 19% for SKOV3ip1 (*p* = 0.004) models (Fig. 4D,E).

**Figure 5:**
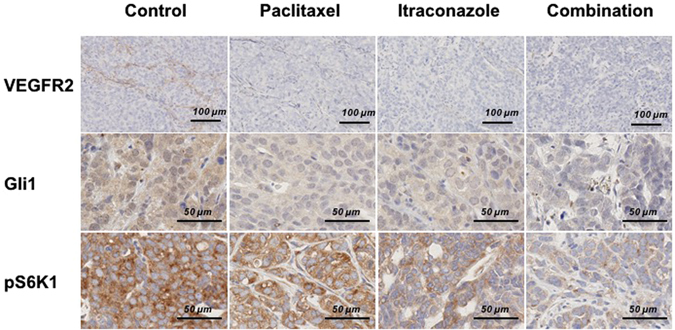
Itraconazole treatment decreases expression of key signaling molecules in the VEGFR2, hedgehog, and mTOR signaling pathways. Inhibition of VEGFR2, hedgehog, and mTOR pathways, as assessed by evaluating expression of VEGFR2, Gli1, and pS6K1 based on immunohistochemical staining of harvested tumor tissues, was significantly lower in the combination group than the paclitaxel alone group. All photographs were taken at a magnification of  x100.

To validate the results of *in vitro* studies, markers of hypoxia, vascularity, hedgehog signaling, and mTOR pathway were assessed using immunohistochemical staining. Expression of VEGFR2 (angiogenesis), Gli1 (hedgehog signaling), and S6K1 (mTOR pathway) were decreased in tumors treated with itraconazole (Fig. 5). The combination of itraconazole and paclitaxel further decreased the expression levels of these molecules.

### Itraconazole and paclitaxel have a synergistic effect on inhibiting tumor growth in EOC PDX models

We also examined the effects of itraconazole in PDX models of serous adenocarcinoma and carcinosarcoma using subrenal implantation of human EOC tissue. We selected case number OV20, a serous adenocarcinoma, and OV22, a carcinosarcoma, which is known to be a chemoresistant tumor^12^. OV20 case was a 71-year-old patient with FIGO stage IIIC. She was treated with primary cytoreduction followed by paclitaxel-carboplatin combination chemotherapy. The residual tumor after surgery was stable during chemotherapy, but progressed 17 months following chemotherapy. OV22 was a 57-year-old patient with stage II disease.

**Figure 6:**
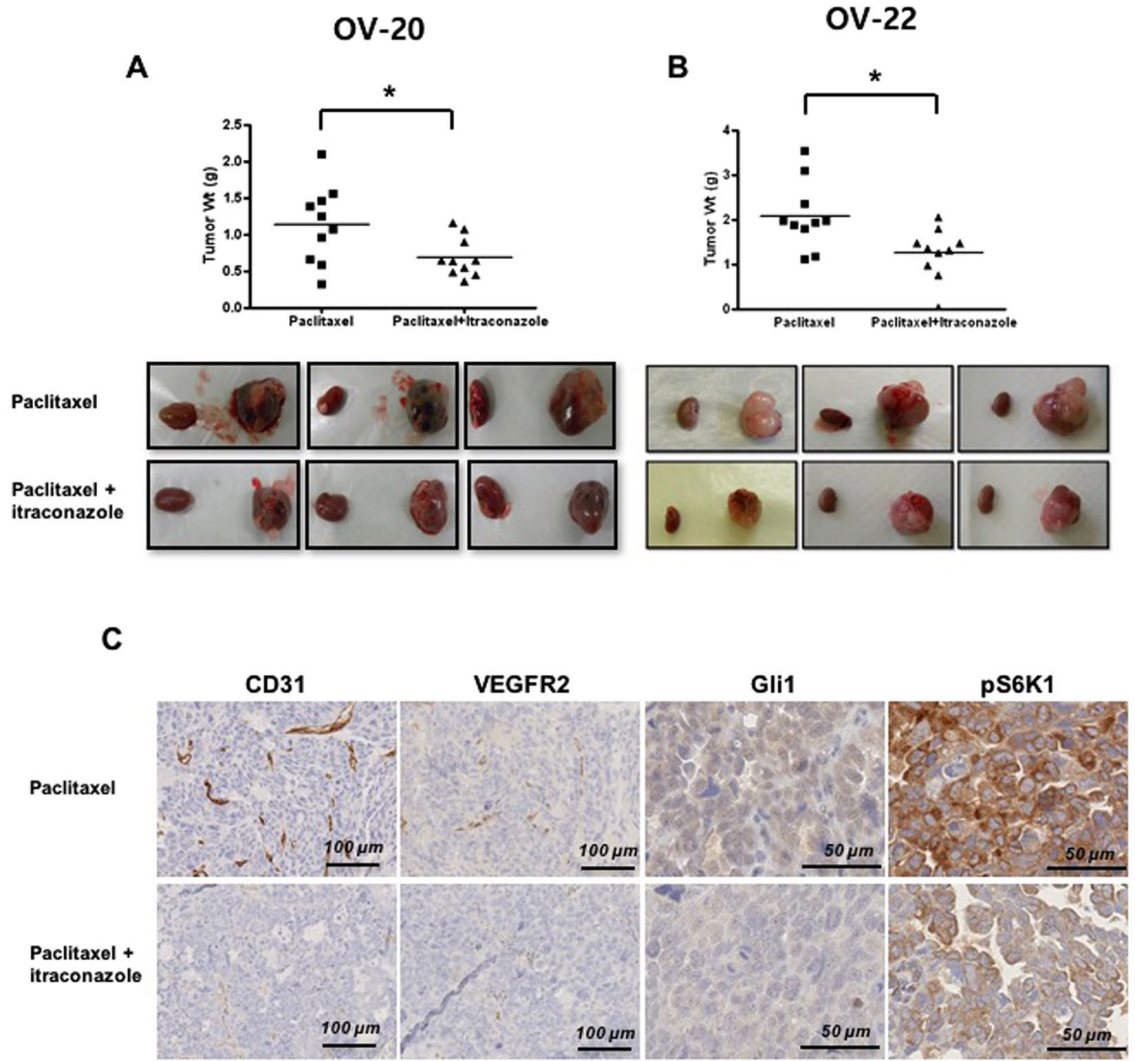
Effects of itraconazole in patient-derived tumor xenograft (PDX) models of serous carcinoma and carcinosarcoma. Itraconazole significantly decreased tumor weight compared with paclitaxel (**A** and **B**). Inhibition of VEGFR2, hedgehog, and mTOR pathways as assessed by significantly decreased immunohistochemistry staining of CD31, VEGFR2, Gli1, and pS6K1 in the combined treatment group (**C**). In each figure, the small left piece is normal kidney (no tumor transplanted), and the right large piece is the developed PDX.

The PDX model was treated for 3 weeks, beginning 5 weeks after implantation of xenograft tissues (passage 3). Addition of itraconazole to paclitaxel significantly enhanced efficacy in this model compared with therapy involving paclitaxel alone (*P* = 0.029 for OV 20 and *P* = 0.019 for OV22, respectively) (Fig. 6). Immunohistochemical staining of the markers yielded similar results to those obtained for the xenograft model. Expression of CD31 and VEGFR2 (angiogenesis), Gli1 (hedgehog signaling), and S6K1 (mTOR pathway) were decreased in tumors treated with combination of paclitaxel and itraconazole.

## Discussion

Here we described the results of a series of *in vitro* and *in vivo* analyses evaluating the antiangiogenic activities of itraconazole and its possible synergistic effect with paclitaxel. In the *in vitro* study, itraconazole significantly inhibited the proliferation of endothelial cells in a dose-dependent manner, but failed to alter the proliferation of EOC cells. In orthotopic mouse models, mice treated with the combination of itraconazole and paclitaxel had significantly decreased tumor weight compared to the other groups. These results were corroborated in PDX animal models derived from patients with a chemoresistant serous carcinoma or carcinosarcoma; the combination of itraconazole and paclitaxel significantly reduced tumor growth compared to paclitaxel alone. These results suggest that itraconazole selectively inhibits endothelial cells rather than cancer cells themselves by targeting multiple pathways including angiogenesis, hedgehog, and mTOR pathways. Adding itraconazole to paclitaxel chemotherapy could potentially enhance the drug response in patients with EOCs.

Despite the failure to alter the proliferation of EOC cells with itraconazole *in vitro*, in orthotopic mouse models, mice treated with the combination of itraconazole and paclitaxel had significantly decreased tumor weight compared to the other groups. The inconsistent results between *in vivo* and *in vitro* analysis might be the results of antiangiogenic effect in endothelial cells by itraconazole and cytotoxic effect in EOC cells by paclitaxel, finally resulting in tumor regression. In addition, the synergistic efficacy of paclitaxel and itraconazole may have efficacy in tumor regression. To evaluate the synergistic efficacy of paclitaxel and itraconazole, we evaluated the *in vitro* assay of combination therapy (Supplemental Fig. S1).

Angiogenesis, the formation of new blood vessels, is crucial to the growth and spread of solid tumors, including ovarian cancer^13^. Epithelial ovarian cancer is the leading cause of death from gynecologic cancer and, at the time of diagnosis, has usually spread throughout the abdominal cavity, followed by growth factor-modulated angiogenesis. VEGF expression is a prognostic marker in ovarian cancer^14, 15^. Several antiangiogenic agents have been demonstrated to result in improved outcomes in randomized phase III trials. However, despite initial optimism, the results of trials have largely been disappointing. The survival benefit associated with antiangiogenic agent is marginal, and a cost-effectiveness analysis showed that, given their limited efficacy and significant toxicity, the cost could be considered excessive^16^. These observations support further development of novel anti-angiogenic strategies for patients with ovarian cancer, including agents with mechanisms of action distinct from currently available monoclonal antibodies and receptor tyrosine kinases (RTKs).

Itraconazole is an orally bioavailable antifungal agent that inhibits the enzyme lanosterol 14a-demethylase. Chong *et al*. initially reported that itraconazole inhibited endothelial cell proliferation and blocked several growth factors involved in angiogenesis^4^. We demonstrated in this study that the antiangiogenic activity of itraconazole is attributable to its ability to inhibit the VEGFR2, hedgehog, and mTOR pathways. Treatment with itraconazole significantly decreased the expression of VEGFR2 and phosphorylation of ERK and phospholipase C gamma 1/2 (PLCr1), a direct binding partner of VEGFR2 in endothelial cells. The expression of Gli1, Ptch1, and SMO, markers of the hedgehog pathway, was also inhibited by itraconazole. In addition, the phosphorylation state of S6 kinase, a canonical mTOR substrate, was inhibited by itraconazole. Although conventional angiogenesis inhibitors are available in the clinic for EOC, their efficacy is modest due in part to the existence of alternative and compensatory signaling pathways. This study shows that itraconazole has potential anti-angiogenic effects, which is important for the prevention of chemoresistance.

In EOC, a combination of agents is important to ensure that chemotherapy is efficacious and to prevent chemo-resistance^17^. Itraconazole had synergistic effects when used in combination with paclitaxel-based chemotherapy, one of the most effective agents available to treat EOC. The greatest effect was observed for endothelial cells and suggests the prevention of acquired resistance. In recent studies, several combinations of itraconazole were shown to be effective against various cancer types^8, 18, 19^.

Typical tissue culture conditions, including high oxygen tension, high glucose concentration, and low hydrostatic pressure, are different from the biologic conditions in which tumors naturally arise. We therefore employed an alternative approach based on primary ovarian cancer xenografts. The primary xenograft model depends on immediate transfer of human cancers from patients into recipient mice without intervening tissue culture or cell line derivation *ex vivo*.

Oxidative stress results in an accumulation of reactive oxygen species (ROS) thus promoting leukocyte adhesion and increasing endothelial permeability. The resulting chronic injury stimulus results in progressive cellular hypometabolism, which appears to be a central initiating factor for vascular abnormalities and mitochondrial damage^20^. Mitochondria are the predominant source of reactive oxygen species (ROS) in the cell^21^. It is estimated that 90% of cellular oxygen is consumed in the mitochondria and about 2–4% of that oxygen is converted to ROS. These studies emphasizes the importance of mitochondrial control of Nox1 redox signaling, an important factor for designing possible therapeutic strategies against cancer^22^.

In this paper we have demonstrated the synergy of a standard of care chemotherapeutic with the addition of widely used antifungal agents to enhance efficacy in xenograft and PDX models. Re-purposing of clinical drugs can allow for more rapid and cost effective transition to clinical studies compared to new drug entities. In conclusion, our findings have important clinical implications for administration of itraconazole as a novel antiangiogenic agent for treatment of EOC. Systemically administered itraconazole showed synergistic effects when combined with paclitaxel with regard to inhibiting the growth of ovarian cancer in a mouse allograft model and did so at serum levels comparable to those in patients undergoing antifungal therapy. Clinical trials evaluating the combination of paclitaxel and itraconazole are needed in the future.

## Materials and Methods

### Chemicals and cell culture

Itraconazole was obtained from Sigma-Aldrich Inc. (St. Louis, MO), and Sporanox (Itraconazole: oral solution) was purchased from Janssen-Clag Ltd. (Batch NO. BKB4000). Endothelial cell lines (HUVEC and SVEC4-10) were obtained from Gibco (Invitrogen, Carlsbad, CA92008, USA) and the American Type Culture Collection (ATCC, Manassas, VA, USA). SKOV3ip1 and HeyA8 were a gift from Dr. Anil K. Sood, Department of Cancer Biology, University of Texas M.D. Anderson Cancer Center, TX, USA. All ovarian cancer cells (SKOV3ip1 or HeyA8) were maintained in RPMI 1640 supplemented with 10% fetal bovine serum (FBS). HUVECs were maintained in M200 media, and SVEC4-10 (mouse endothelial) cells were maintained in DMEM media supplemented with 10% fetal bovine serum (FBS). All cells were maintained in 5% CO_2_ at 37 °C.

### Cell proliferation assay

Cells were plated in culture medium in a 96-well plate at 3 × 10^3^ cells/well. After 24 h, cells were treated with itraconazole, and the assay was performed at 48 h. For proliferation assays, cells were stained with 3-(4,5-dimethylthiazol-2-yl)-2,5-diphenyltetrazolium bromide (MTT; Amresco, Solon, OH, USA); after 4 h of additional incubation, the medium was discarded, 100 *μ*l of acidic isopropanol (0.1 N HCL in absolute isopropanol) was added, and the plate was shaken gently. Absorbance was measured on an enzyme linked immunosorbent assay (ELISA) reader at a wavelength of 540 nm.

### Apoptosis assay

Cell apoptosis was measured at 48 h after treatment using the Annexin VFITC apoptosis Detection Kit-1 (BD Pharmingen, San Diego, CA) according to the manufacturer’s protocol. Each sample was assayed in triplicate. A minimum of 5,000 cells were then analyzed by FACScan with Cell Quest software (Backton Dickinson) for acquisition and analysis.

### Western blot

Cells were lysed in PRO-PRE Protein Extraction Solution (Intron Biotechnology, Seongnam, Korea). Protein concentration was determined using a Bradford assay kit (BIO-RAD, Hercules, CA). Cell lysates (50 *μ*g of total protein) were separated in 8% acrylamide gels by sodium dodecyl sulfate-polyacrylamide gel electrophoresis (SDS-PAGE) and transferred to Hybond-ECL nitrocellulose filter paper (Amersham Biosciences, Buckinghamshire, UK). Membranes were blocked with 5% skim milk in Tris-buffered saline containing 0.1% Tween-20 for 1 h at room temperature. Protein bands were probed with VEGFR2 antibody (Santa Cruz Biotechnology, USA) at a 1:500 dilution; total-S6K1, and phospho-S6K1 (p-S6K1) (Santa Cruz Biotechnology, USA), total-Erk (Thr202/Tyr204), phosphor-Erk (Thr202/Tyr204), total-PLCr 1/2, phospho-PLCr 1/2 (Cell Signaling, USA), Gli 1, SMO, and PTCH 1 antibodies (Abcam, England) at 1:1000 dilutions; *β*-actin antibody at a 1:4000 dilution (Santa Cruz Biotechnology, Santa Cruz, USA); and *α*-tubulin antibody at a 1:3000 dilution (Epitomics, Burlingame, USA) and then labeled with horseradish peroxidase-conjugated anti-rabbit antibody (GE Healthcare, Piscataway, USA). Bands were visualized by enhanced chemiluminescence using an ECL kit (Amersham Biosciences, Buckinghamshire, UK) according to the manufacturer’s protocol.

To examine the change of oxidative stress marker, we evaluated the protein expression of SOD2 and catalase. Cells were treated with itraconazole (1 or 1.5 µM) for 1, 6 and 24 h.whole cell lysates were analyzed by western blotting using antibodies against oxidative stress markers such as SOD2 and catalase.

### Semi-quantitative RT-PCR

The cDNA was amplified by PCR using the following primer sequences for *Gli1*: Human (H)-*Gli1*-5′-CAGGGAGTGCAGCCAATACAG-3′(forward) and 5′-GAGCGGCGGCTGACAGTATA-3′ (reverse) and mouse (M) M-*Gli1*-5′-CGACCGAAGGTGCGTCTTGAG-3′(forward) and 5′-AGCCCTGGACCACGCATC-3′ (reverse). Beta-actin was amplified from cDNA as an endogenous control using the following primers: 5′-GATGCAGAAGGAGATCACTG-3′ (forward) and 5′-AGTCATAGTCCGCCTAGAAG-3′ (reverse). PCR was carried out by performing an initial denaturation step at 95 °C for 10 min, followed by 35 cycles for *Gli1* and 28 cycles for *ACTB* of denaturation (95 °C, 40 sec), annealing (58 °C for *Gli1*, 57 °C for β-actin, 40 sec), and extension (72 °C, 30 sec). This was followed by a final extension step of 72 °Cfor 5 min. Amplification products were electrophoresed on a 1.2% agarose gel and visualized by ethidium bromide staining under ultraviolet transillumination. Images of RT-PCR ethidium bromide-stained agarose gels were acquired with a Cohu High Performance CCD camera (Cohu Inc. San Diego, CA) and quantification of the bands was performed by Phoretix 1 D (Phoretix International Ltd., Newcastle upon Tyne, UK). Band intensity was expressed as relative absorbance units.

### Reactive Oxygen species (ROS) expression Assay

To image intracellular ROS, HUVEC, SVEC4-10 and SKOV3ip1 cells were plated on 2 well chamber slides. After 16 h, cells were rinsed before being loaded with 2 uM CM-H2DCFDA in HBSS for 30 min at 37 °C. Buffer was changed to culture media with or without 1 µM or 1.5 µM itraconazole, after which cells were incubated for 30 min at 37 °C. After treatment, the chamber slides were mounted onto a cover glass with Gel/Mount™. Imaging was performed on a fluorescence microscope using a filter for FITC fluorescence at x200 magnification.

### Animal care and development of ***in vivo*** models including established cell lines and PDX

*In vivo* experiments were performed to confirm the synergistic effects of itraconazole and paclitaxel on tumor growth in orthotopic cell-lines or patient-derived xenograft (PDX) mouse models. Female BALB/c nude mice were purchased from ORIENT BIO (Sungnam, Korea). This study was performed in accordance with relevant guidelines and regulations. This study was reviewed and approved by the Institutional Animal Care and Use Committee (IACUC) of Samsung Biomedical Research Institute (SBRI). SBRI is an Association for Assessment and Accreditation of Laboratory Animal Care International (AAALAC International, protocol No. H-A9-003)-accredited facility and abides by the Institute of Laboratory Animal Resources (ILAR) guidelines.

To generate tumors, SKOV3ip1 (1.0 × 10^6^ cells/0.2 mL HBSS) and/or HeyA8 (2.5 × 10^5^ cells/0.2 mL HBSS) were injected into the peritoneal cavity of BALB/c nude mice, that were 8 to 12 weeks old^23^. To generate PDX models of EOC, two patient tumor specimens retrieved from the operation room were cut into small pieces (below 2–3 mm), implanted into the subrenal capsule of the left mouse kidney^24, 25^ and propagated by serial transplantation. Tumors were derived from one patient with serous adenocarcinoma of FIGO stage IIIC (OV-20) and one patient with carcinosarcoma of the ovary (OV-22). After 7 days of cell injection for the cell line models or 5 weeks for the PDX models, paclitaxel (6 mg/kg) or PBS was injected i.p. once weekly, and oral itaconazole 20 mg/kg was administered twice daily in a 200 *μ*l volume.

Four groups of xenograft mice (*n* = 10 per group) for each cell line and two groups for PDX (*n* = 10 per group) were monitored for adverse effects. Mice were monitored daily for tumor development and postoperative complications and were sacrificed on days 35 to 40 or if mice seemed moribund. Total body weight and tumor weight of each mouse were recorded. Tumors were fixed in formalin and embedded in paraffin or snap frozen in OCT compound (Sakura Finetek Japan, Tokyo, Japan) in liquid nitrogen.

### Immunohistochemical analysis

Immunohistochemical staining was performed with the standard peroxidase/DAB method (Dako, Carpinteria, CA) on 5-μm-thick formalin-fixed, paraffin-embedded tissue sections. Tissue sections were deparaffinized with xylene and dehydrated through a graded ethanol series. Endogenous peroxidase activity was quenched with 3% H_2_O_2_ in water for 15 min. Sections were subjected to antigen retrieval and incubated with primary antibodies, a detailed list of which, along with dilutions, is provided in Supplementary Table S1. Antigen-antibody reactions were detected with the Dako EnVision + System- HRP (Dako) and DAB + (3.3′-diaminobenzidine: Dako) according to the manufacturer’s instructions. Appropriate controls using an isotype-matched antibody instead of the primary antibody were performed. Slides were counterstained with hematoxylin and cover-slipped. Two pathologists blindly reviewed slides and evaluated the immunohistochemical staining.

### Data analysis

The Mann–Whitney U test was used to evaluate the significance of differences among groups for both *in vitro* and *in vivo* assays. All statistical tests were two-sided, and *p* values less than 0.05 were considered to be statistically significant. SPSS software (version 17.0; SPSS, Chicago, IL, USA) was used for statistical analyses.

To determine why paclitaxel and itraconazole functioned synergistically, we assessed the correlation between genes targeted by paclitaxel (tubulin B) and itraconazole (VERFR2, ERK, PLCr1/2, Gli1, Ptch1, SMO, and S6K1). For cancer tissues, data from The Cancer Genome Atlas (TCGA) Research Network (pan-cancer normalized form of RNA‐seq data of ovarian cancers, version: 2015-02-24) were analyzed (http://cancergenome.nih.gov/). For human endothelial cells, GEO data, including those for 38 human endothelial cells, were downloaded (http://www.ncbi.nlm.nih.gov/geo/query/acc.cgi?acc = GSE43475). Interestingly, the correlation was more pronounced in endothelial cells than in ovarian cancer tissues, supporting the role of itraconazole in endothelial cells (Supplementary Fig. S3).

## Electronic supplementary material

Supplementary
