# Supplementary material for: The anti-cancer effects of itraconazole in epithelial ovarian cancer
Source: Sci Rep. 2017 Jul 26;7:6552. doi: 10.1038/s41598-017-06510-7 (PMC5529373; doi:10.1038/s41598-017-06510-7)
Supplement: Supplementary file 1 — Supplementary [file 41598_2017_6510_MOESM1_ESM.doc]

**The anti-cancer effects of itraconazole in epithelial ovarian cancer**

Chel Hun Choi^1^, Ji-Yoon Ryu^1*^, Young-Jae Cho^1^, Hye-Kyung Jeon^1^, Jung-Joo Choi^1^, Kris Ylaya^2^, , Yoo-Young Lee^1^, Tae-Joong Kim^1^, Joon-Yong Chung^2^, Stephen M. Hewitt ^2^, Byoung-Gie Kim^1^ and Duk-Soo Bae^1^, Jeong-Won Lee^1*^

*Departments of ^1^Obstetrics and Gynecology, Samsung Medical Center, Sungkyunkwan University School of Medicine, Seoul, Korea*

*^2^ Experimental Pathology Laboratory, Laboratory of Pathology, Center for Cancer Research, National Cancer Institute, National Institutes of Health, Bethesda, MD 20892 USA*

**Supplementary Figure S1. Synergistic effect of itraconazole and paclitaxel in endothelial cells.**

Combined treatment of itraconazole and paclitaxel inhibited cell proliferation, as evaluated by the MTT assay in HUVEC (A), SVEC4-10 (B), and SKOV3ip1 (C) cells. Proliferations of endothelial cells were synergistically inhibited, which was not seen in SKOV3ip1 cells.

**
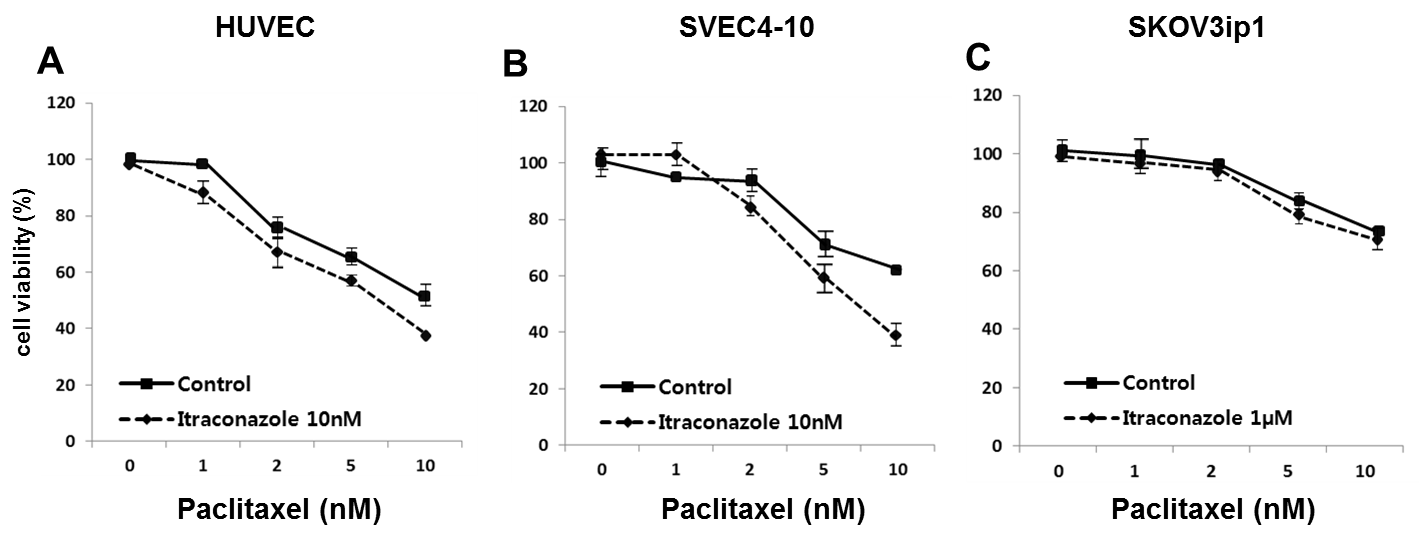
**

**Supplementary Figure S2. Expression of oxidative stress marker**

The expressions of SOD2 and catalase examined using western blotting (A), which showed increased expression of catalase with treatment of itraconazole. Intracellular ROS was examined in HUVEC, SVEC4-10 and SKOV3ip1 cells using fluorescence microscope (B).

**
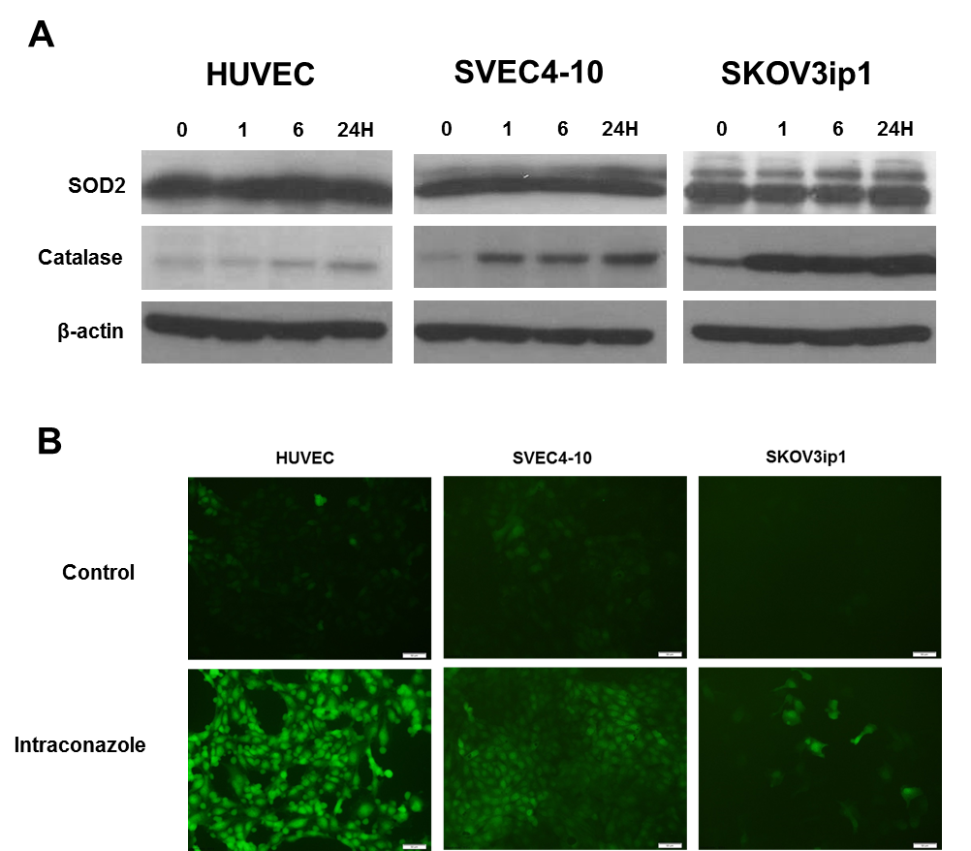
**

**Supplementary Figure S3. Correlation between the targets of paclitaxel and itraconazole.**

Correlations between beta tubulin (target of paclitaxel) and VEGFR2, ERK, PLCG1, PLCG2, Gli1, Ptch1, SMO, and S6K1 (targets of itraconazole) were evaluated. A higher correlation was found for endothelial cells (A) than cancer tissues (B). Plot of correlation coefficient and p value showed a higher correlation in endothelial cells than in TCGA ovarian cancers (C).


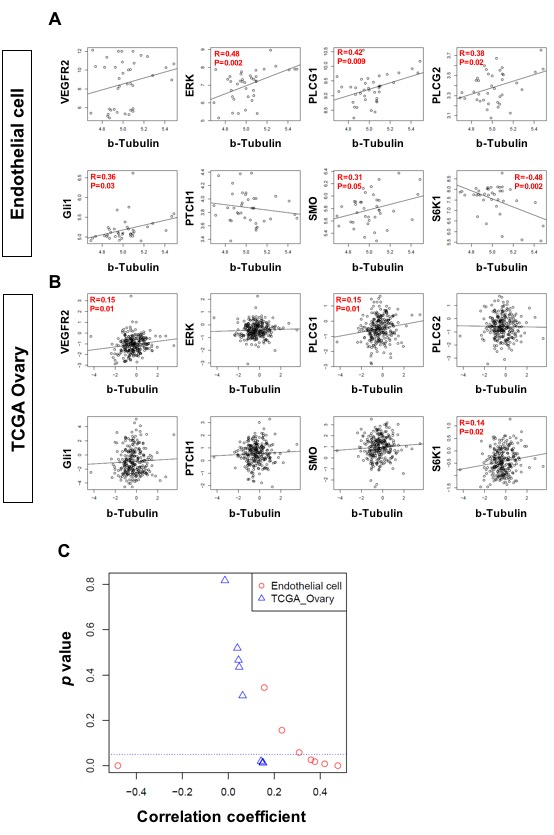


**Supplementary Figure S4. Full-length blots of Fig. 2**


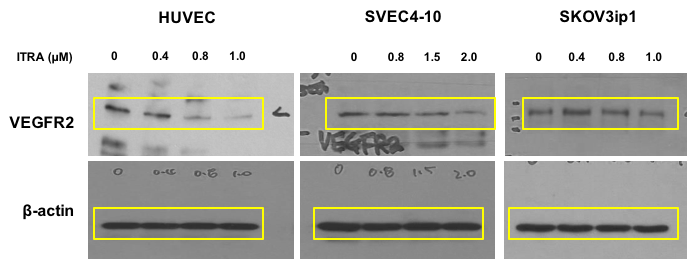


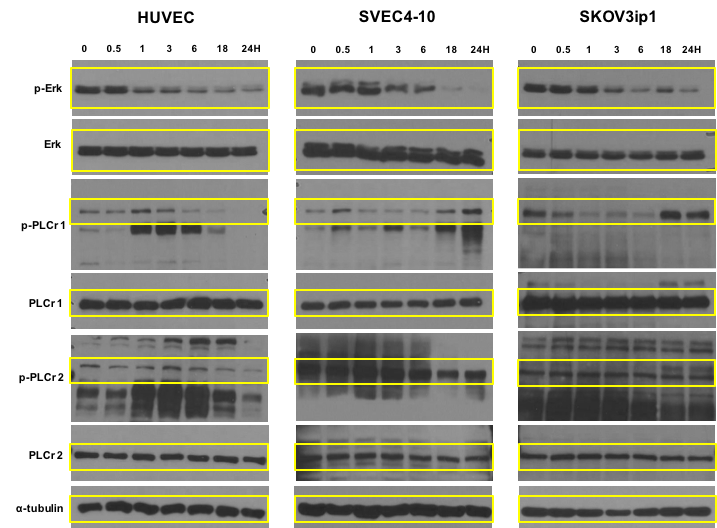


**Supplementary Figure S5. Full-length blots of Fig. 3**


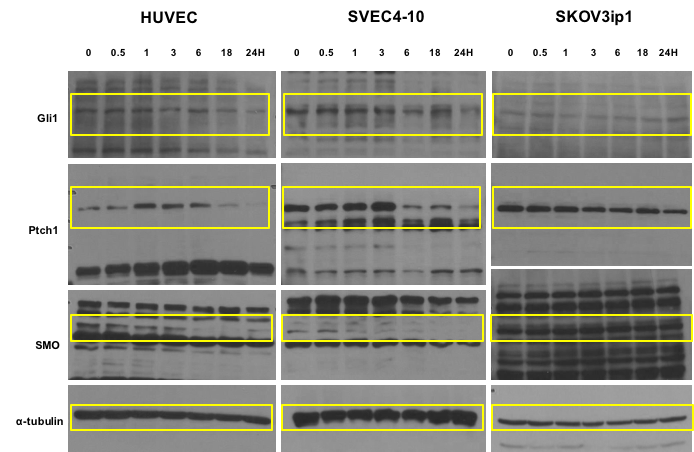


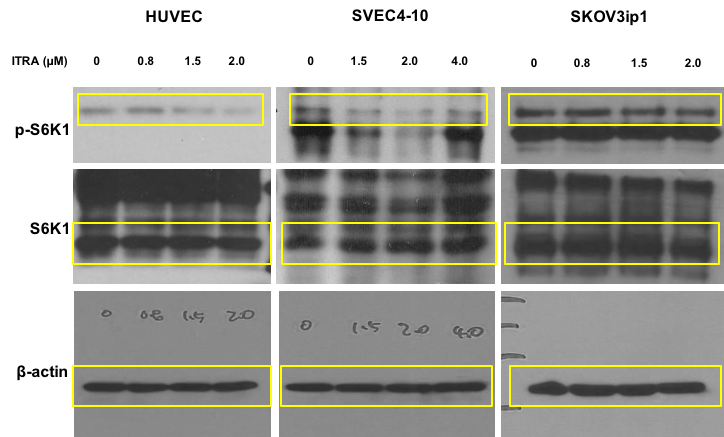


**Supplementary Figure S6. Full-length blots of Supp Fig. S2**


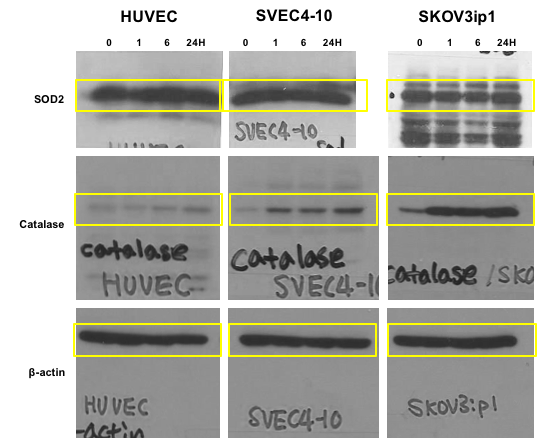


**Supplementary Table 1.** Details of antibodies used for immunohistochemistry.

| Antibody | Vendor | Clonality | Cat. # | Incubation | Dilution | Antigen retrieval |
| --- | --- | --- | --- | --- | --- | --- |
| CD31 | Abcam | Poly rabbit | ab28364 | 60 min at RT | 1/50 | 20 min, pressure  cooker, pH 6 |
| VEGFR2 | Cell Signaling | Mono rabbit (55B11) | #2479 | 60 min at RT | 1/500 | 20 min, pressure  cooker, pH 9 |
| Gli1 | Abcam | Poly rabbit | ab92611 | 120 min at RT | 1/100 | 20 min, pressure  cooker, pH9 |
| pS6K1 | Abcam | Poly rabbit | ab129230 | 60 min at RT | 1/300 | 20 min, pressure  cooker, pH 6 |

RT, room temperature
